# Prediction of blood–brain barrier penetrating peptides based on data augmentation with Augur

**DOI:** 10.1186/s12915-024-01883-4

**Published:** 2024-04-19

**Authors:** Zhi-Feng Gu, Yu-Duo Hao, Tian-Yu Wang, Pei-Ling Cai, Yang Zhang, Ke-Jun Deng, Hao Lin, Hao Lv

**Affiliations:** 1 The Clinical Hospital of Chengdu Brain Science Institute, School of Life Science and Technology, University of Electronic Science and Technology of China, Chengdu, 610054 PR China; 2 Center for Informational Biology, School of Life Science and Technology, University of Electronic Science and Technology of China, Chengdu, 611731 PR China; 3 School of Basic Medical Sciences, Chengdu University, Chengdu, 610106 PR China; 4 Innovative Institute of Chinese Medicine and Pharmacy, Academy for Interdiscipline, Chengdu University of Traditional Chinese Medicine, Chengdu, 610072 PR China

**Keywords:** Blood–brain barrier, Penetrating peptides, Machine learning, Data augmentation, Feature selection, Information gain

## Abstract

**Background:**

The blood–brain barrier serves as a critical interface between the bloodstream and brain tissue, mainly composed of pericytes, neurons, endothelial cells, and tightly connected basal membranes. It plays a pivotal role in safeguarding brain from harmful substances, thus protecting the integrity of the nervous system and preserving overall brain homeostasis. However, this remarkable selective transmission also poses a formidable challenge in the realm of central nervous system diseases treatment, hindering the delivery of large-molecule drugs into the brain. In response to this challenge, many researchers have devoted themselves to developing drug delivery systems capable of breaching the blood–brain barrier. Among these, blood–brain barrier penetrating peptides have emerged as promising candidates. These peptides had the advantages of high biosafety, ease of synthesis, and exceptional penetration efficiency, making them an effective drug delivery solution. While previous studies have developed a few prediction models for blood–brain barrier penetrating peptides, their performance has often been hampered by issue of limited positive data.

**Results:**

In this study, we present Augur, a novel prediction model using borderline-SMOTE-based data augmentation and machine learning. we extract highly interpretable physicochemical properties of blood–brain barrier penetrating peptides while solving the issues of small sample size and imbalance of positive and negative samples. Experimental results demonstrate the superior prediction performance of Augur with an AUC value of 0.932 on the training set and 0.931 on the independent test set.

**Conclusions:**

This newly developed Augur model demonstrates superior performance in predicting blood–brain barrier penetrating peptides, offering valuable insights for drug development targeting neurological disorders. This breakthrough may enhance the efficiency of peptide-based drug discovery and pave the way for innovative treatment strategies for central nervous system diseases.

## Background

Central nervous system (CNS) injury is a significant factor in disability. However, treating neurological diseases is challenging as the blood–brain barrier (BBB) blocks almost all macromolecular drugs and 98% of small molecule drugs [1]. In recent years, peptide-based drug delivery carriers have gained prominence as a novel approach for the diagnosis and treatment of brain disorders, owing to their higher biocompatibility, which effectively overcomes the immunogenicity and high production costs with traditional protein carriers [1]. Among them, blood–brain barrier penetrating peptides (B3PPs) have emerged as ideal drug delivery carriers [2, 3], as they navigate the BBB through their endogenous mechanism [4, 5], facilitating the entry of small-molecule drugs into the CNS.

To date, several experimental techniques—such as the phage display method [6, 7], the retro-enantio approach [8], and radionuclide labeling—have been developed to detect B3PPs [9]. However, traditional experimental pipelines are inefficient and costly [10]. Therefore, it is necessary to introduce computational methods to improve the efficiency of identifying B3PPs, thereby promoting the discovery of peptide drugs.

During the past few years, several computational models have been proposed to identify B3PPs. For instance, Dai et al. designed a feature representation learning strategy to characterize sequence-based features from a wide variety of feature descriptors [11–13]. Zou developed a B3PP identification method based on amino acids physicochemical properties [14, 15], Pearson’s correlation coefficient, and maximal information coefficient. In addition, Kumar et al. proposed another computational tool-based machine learning [16], called B3Pred, for B3PPs identification. Recently, He et al. developed a novel meta-learning-based prediction model called MIMML for bioactive peptide discovery [17]. Charoenkwan et al. built an efficient scoring card method-based predictor (termed SCMB3PP) for improving B3PPs identification and characterization [18].

**Fig. 1:**
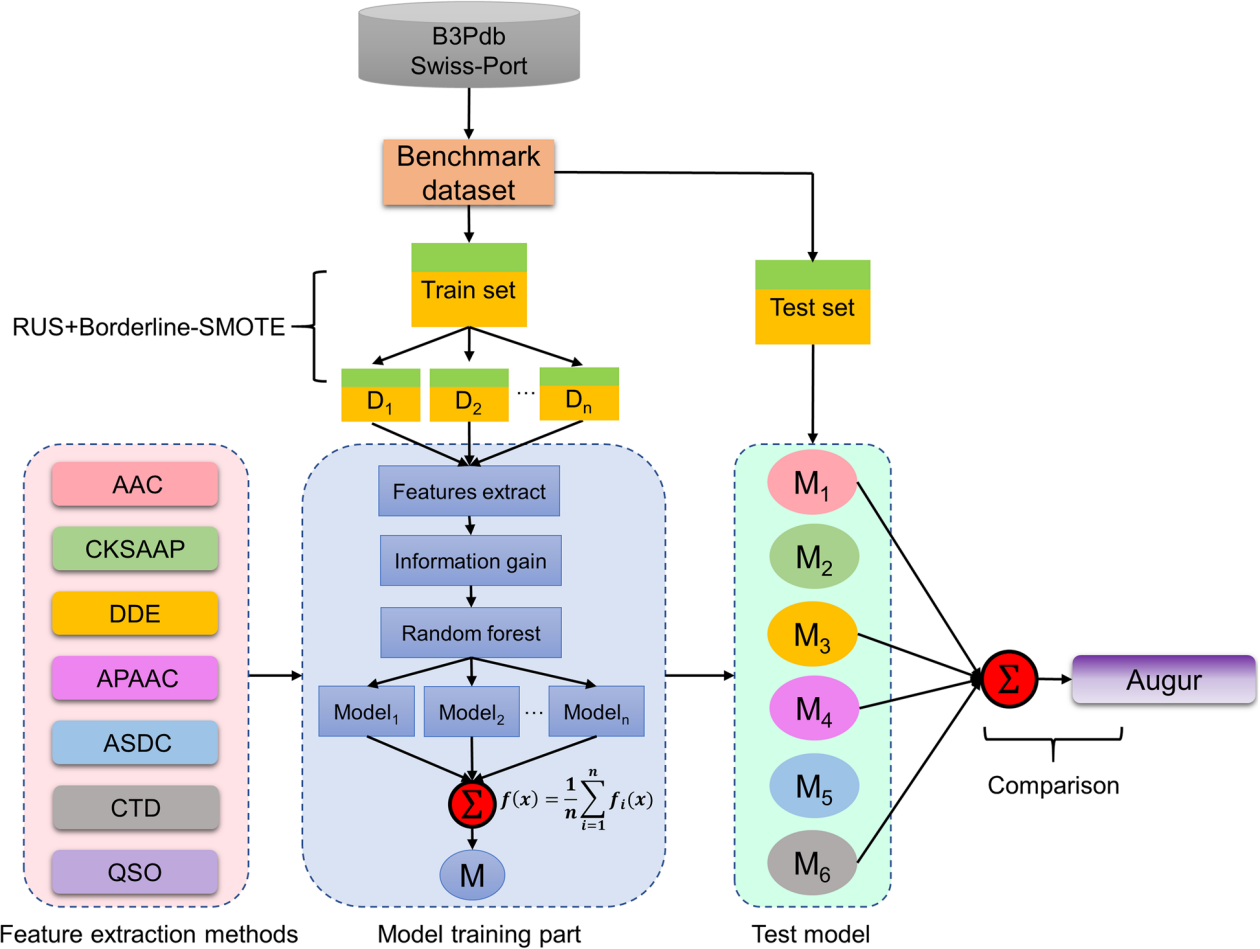
The schematic framework of Augur for B3PPs prediction

Although previous models have made significant contributions to the prediction of B3PPs, several issues remain. First, limited small-scale datasets may lead to overfitting and weak generalization ability of prediction models. In addition, an unbalanced ratio of positive and negative samples may be related to a bias in model performance. In this study, we propose data augmentation-based machine learning (ML) model called Augur, which extracts highly interpretable physicochemical properties of B3PPs while solving the issues of small sample size and imbalance of positive and negative samples. Experimental results demonstrate the superior prediction performance of Augur with an AUC value of 0.932 on the training set and 0.931 on the independent test set. The schematic framework of Augur for B3PPs prediction is shown in Fig. 1.

## Results

### Amino acid composition analysis

**Fig. 2:**
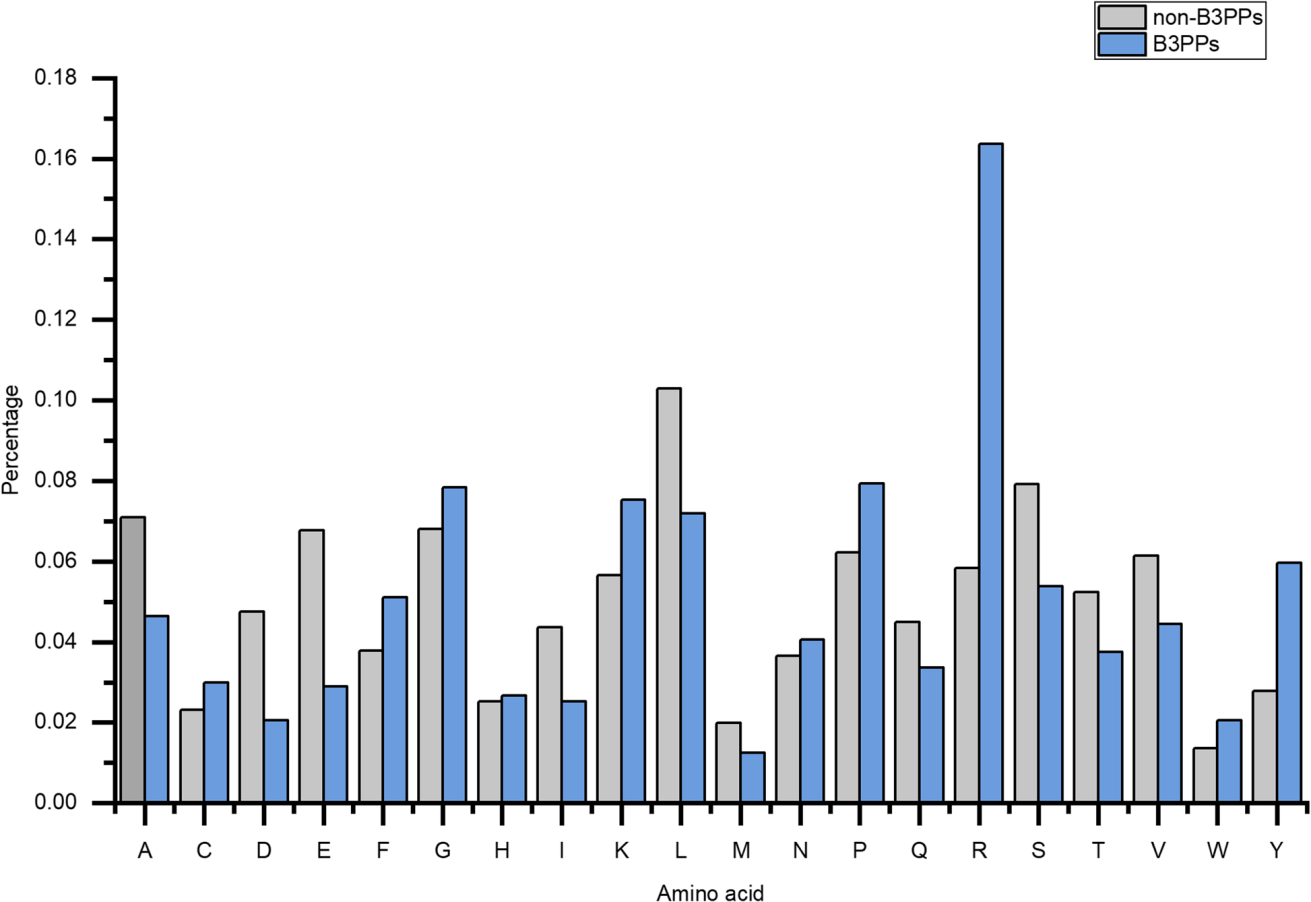
A bar graph to represent percentage amino acid composition of B3PPs and random peptides

**Fig. 3:**
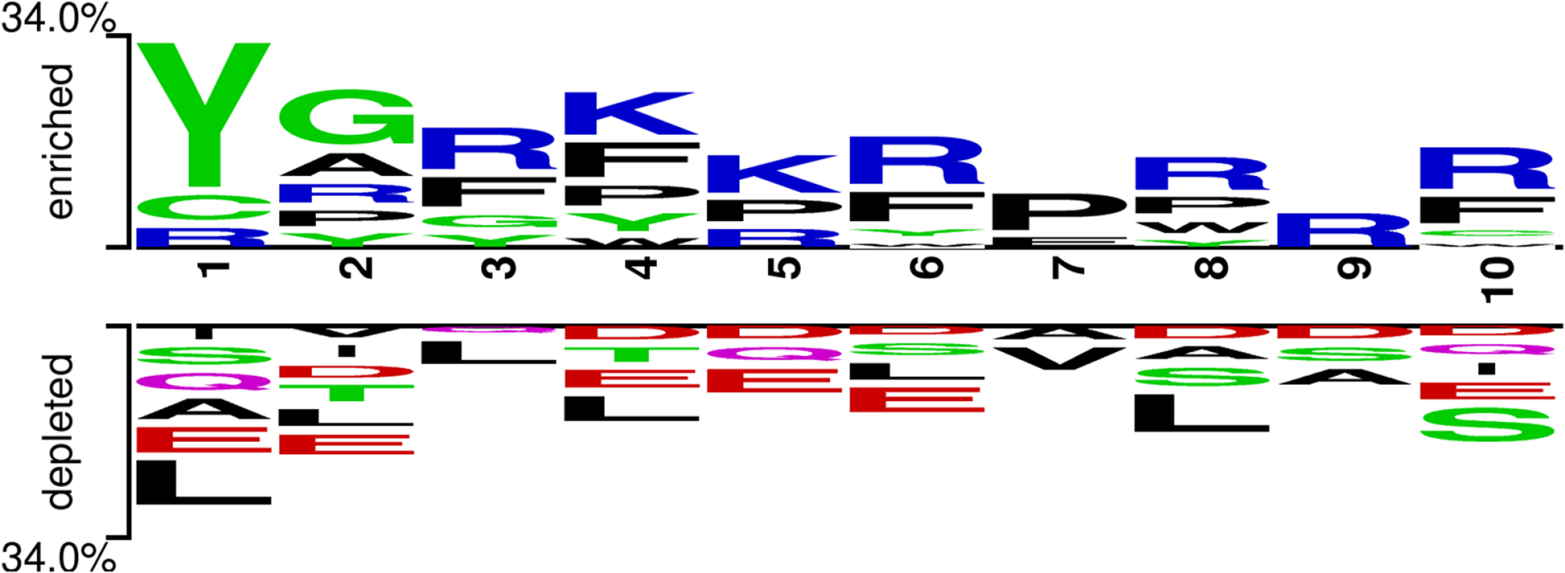
The amino acid position preference of B3PPs

To determine the key amino acids and their distribution in B3PPs, we analyzed the distribution differences of 20 amino acids between B3PPs and non-B3PPs sequences. The bar chart in Fig. 2 revealed that there are some significant differences in the content of certain amino acids. Notably, the arginine (R) content in B3PPs was significantly higher than that of any other amino acid. The contents of glycine (G), lysine (K), leucine (L), and proline (P) were not only similar to each other but also higher than the rest of the amino acids. In addition, arginine (R) and tyrosine (Y) are significantly enriched in B3PPs. We also used the Two Sample Logo (TSL) to examine the amino acid position preference in B3PPs, which was shown in Fig. 3. The results showed that B3PPs have high abundance of arginine (R) and tyrosine (Y). We speculate that the negatively charged characteristics of the BBB surface greatly reduce the permeability of the BBB to negatively charged solutes while increasing the permeability to positively charged solutes. It means the positive charge of arginine would be beneficial for B3PPs to penetrate the BBB, making arginine an essential component of B3PPs. These findings are consistent with those of Walter et al. [19]. With respect to tyrosine, however, there is currently no experiment to prove that tyrosine helps peptides penetrate the BBB.

### Performance evaluation of different feature extraction methods

**Fig. 4:**
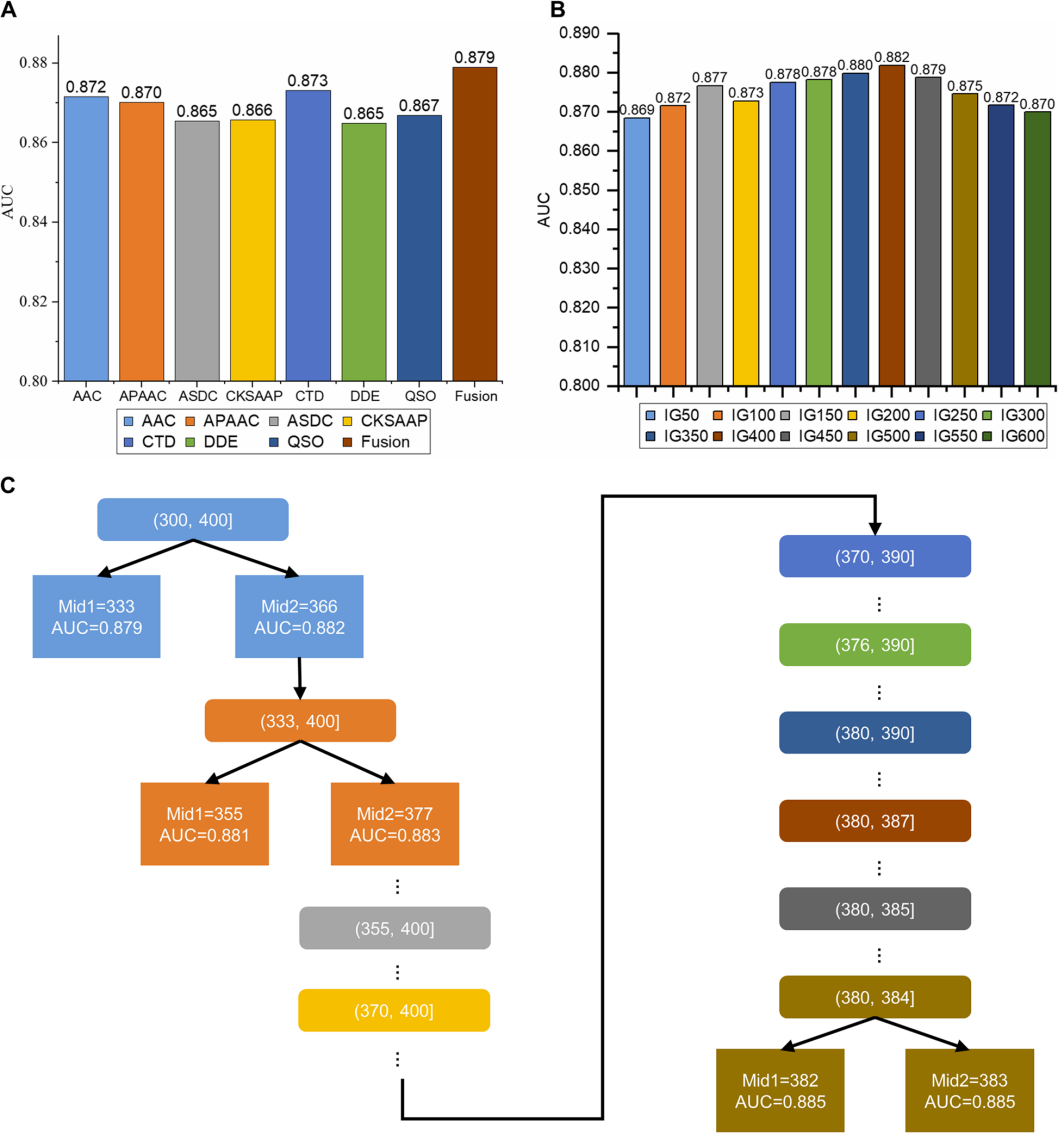
Analysis of single feature, fusion features and optimal feature set. **A** AUCs based on the single feature. **B** AUCs based on the fusion features optimized by IG. **C** Partial process demonstration of the ternary search algorithm

In this study, the performances of seven different feature encoding methods were evaluated based on five-fold cross-validation (Fig. 4A). The results indicated that the single feature encoding method achieved very close predictive performance. To further enhance the predictive performance of the model, the extracted features were combined into a 2761-dimensional feature set. The model trained on the fused feature set (AUROC = 0.890) exhibited better performance than those on single feature encoding (all AUROCs are below 0.881), indicating that feature fusion strategy played a critical role in predicting B3PPs and significantly improved the predictive performance.

However, fusion features may contain a lot of redundant information, leading to a decrease in model performance. Therefore, the IG feature selection method was applied to the full feature set, and the ranked features were obtained. Different feature sets that contained the top-ranked features were then created and tested, ranging from the top 50 features to the top 600 features, with a step size of 50 (Fig. 4B). It was found that as the feature dimension increased, the performance of the model improved. Moreover, when the number of features selected by IG was greater than 400, the performance of the model tends to decrease, which corresponded to an AUROC of 0.882. In addition, we used the ternary search algorithm to select the optimal number of features and finally determined that the model performed best when it reached 383 (Fig. 4C).

**Fig. 5:**
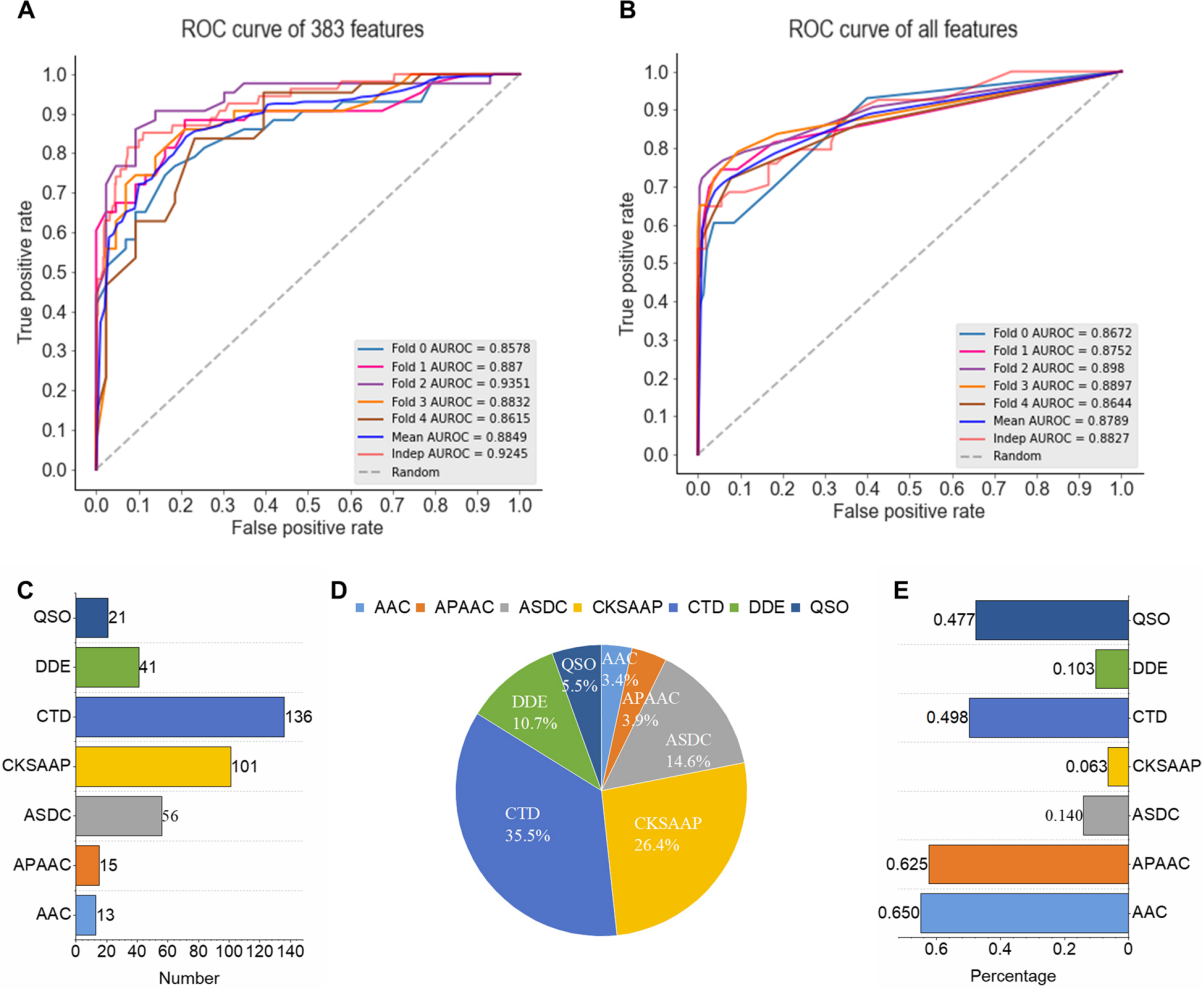
The prediction results using different features. **A** ROC curve of 383 features. **B** ROC curve of all features. **C, D** The number and proportion of the types of features selected in the optimal feature set. **E** The ratio of selected dimension to original dimension in the optimal feature set

Next, we compared the performance of the optimal feature set and the fusion feature set respectively. As shown in Fig. 5A-B, the optimal feature set (AUROC = 0.908) produced better performance in terms of AUROC compared to the fusion features (AUROC = 0.879), indicating that the IG feature selection strategy can effectively filter redundant features and improve the predictive ability of the model. In addition, the feature importance and its contribution were further analyzed to find which feature was more valuable for the model performance after feature selection. As shown in Fig. 5C-E, the optimal feature set contains 35.5% CTD features, 26.4% CKSAAP features, and 14.6% ASDC features, suggesting the significant contribution of these features in the identification of B3PPs. It is worth noting that although the features of AAC and APAAC account for 3.4% and 3.9% respectively in the optimal feature set, these two features are still very valuable based on the ratio of the selected dimension to the original dimension (65% for AAC and 62.5% for APAAC).

### Comparison of different ML methods on B3PPs prediction

**Fig. 6:**
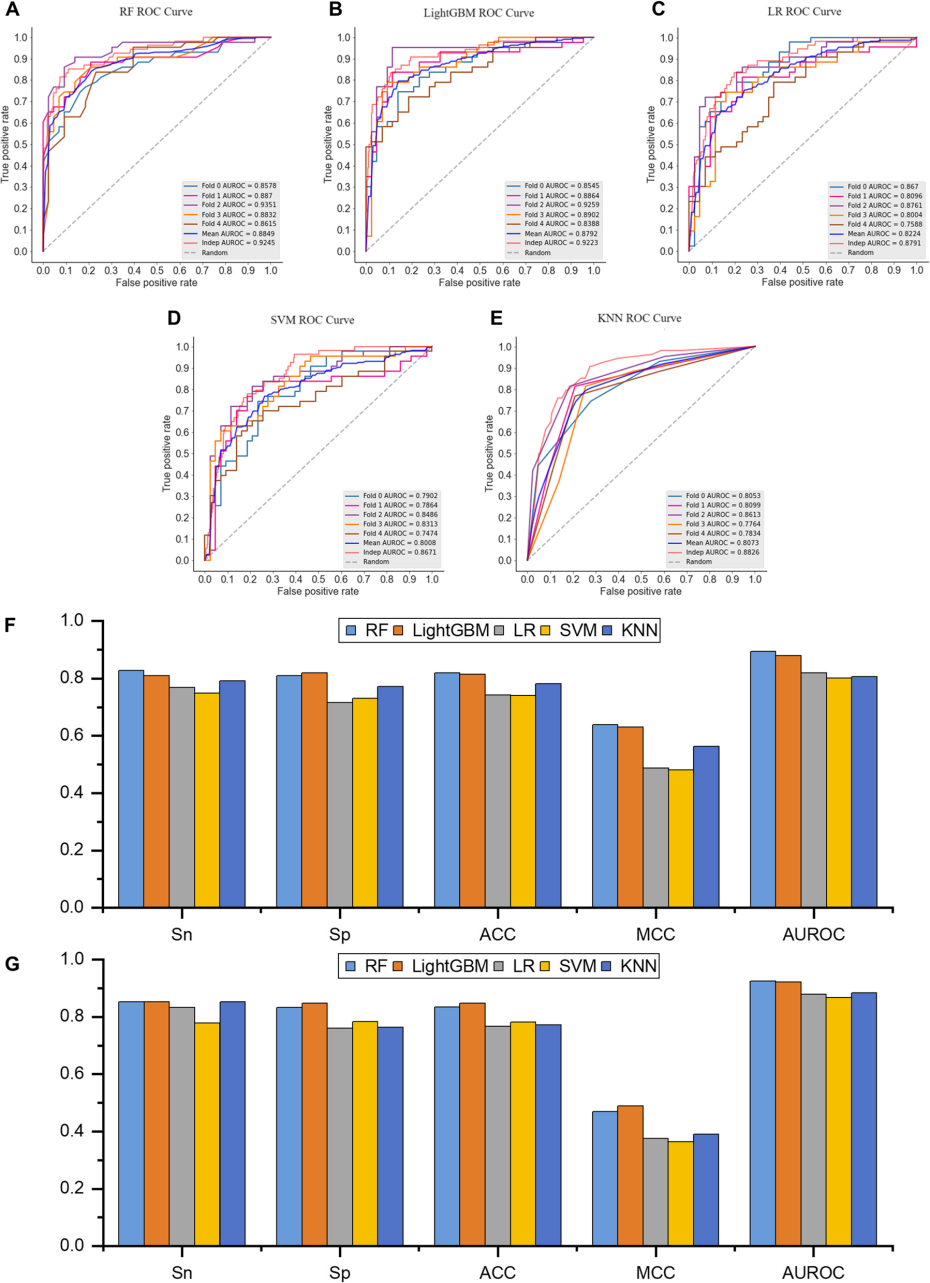
The prediction results using different algorithms. **A** ROC curve of RF. **B** ROC curve of LightGBM. **C** ROC curve of LR. **D** ROC curve of SVM. **E** ROC curve of KNN. **F** Details of evaluation on training set. **G** Details of evaluation on independent test set

To determine optimal ML algorithms for predicting B3PPs, we investigated the discriminant capabilities of RF, LightGBM, LR, SVM, and KNN, on the benchmark dataset by using five-fold cross-validation. The comparisons of the *Sn*, *Sp*, *ACC*, *MCC*, and AUROC among five different ML algorithms are provided in Table 1. The details of evaluation for different algorithms on the training set and the independent test set are shown in Fig. 6. As shown in Fig. 6A-E, the results indicated that there is no significant difference in the predictive performance of the selected machine learning algorithms (AUROC > 0.8). From another perspective, these results also demonstrated that the feature extraction module could learn appropriate feature representations from B3PPs, thereby achieving robust performance regardless of how any machine learning algorithm is ultimately applied. Interestingly, we found that ensemble learning-based algorithms RF and LightGBM achieved leading performance, especially LightGBM yielded competitive prediction capabilities in terms of *Sn*, *Sp*, *ACC*, and *MCC* (Fig. 6F-G). In addition, RF achieved better AUROCs than LightGBM, LR, SVM, and KNN by 0.006, 0.063, 0.084, and 0.078, respectively on the training set. We finally utilized RF to build the prediction model.

**Table 1:** Comparison of multiple ML methods for identifying B3PPs

Evaluation strategy	ML method	*Sn*	*Sp*	*ACC*	*MCC*	AUROC
Training set	RF	0.800	**0.819**	0.809	0.620	**0.885**
	LightGBM	**0.809**	**0.819**	**0.814**	**0.630**	0.879
	LR	0.767	0.716	0.742	0.487	0.822
	SVM	0.749	0.730	0.740	0.481	0.801
	KNN	0.791	0.772	0.781	0.563	0.807
Independent set validation	RF	**0.852**	0.822	0.824	0.454	**0.924**
	LightGBM	**0.852**	**0.848**	**0.848**	**0.489**	0.922
	LR	0.833	0.760	0.767	0.375	0.879
	SVM	0.778	0.783	0.782	0.364	0.867
	KNN	0.852	0.764	0.772	0.390	0.883

Best performance metrics are shown in bold

### Comparison of data augmentation results with different proportions

**Fig. 7:**
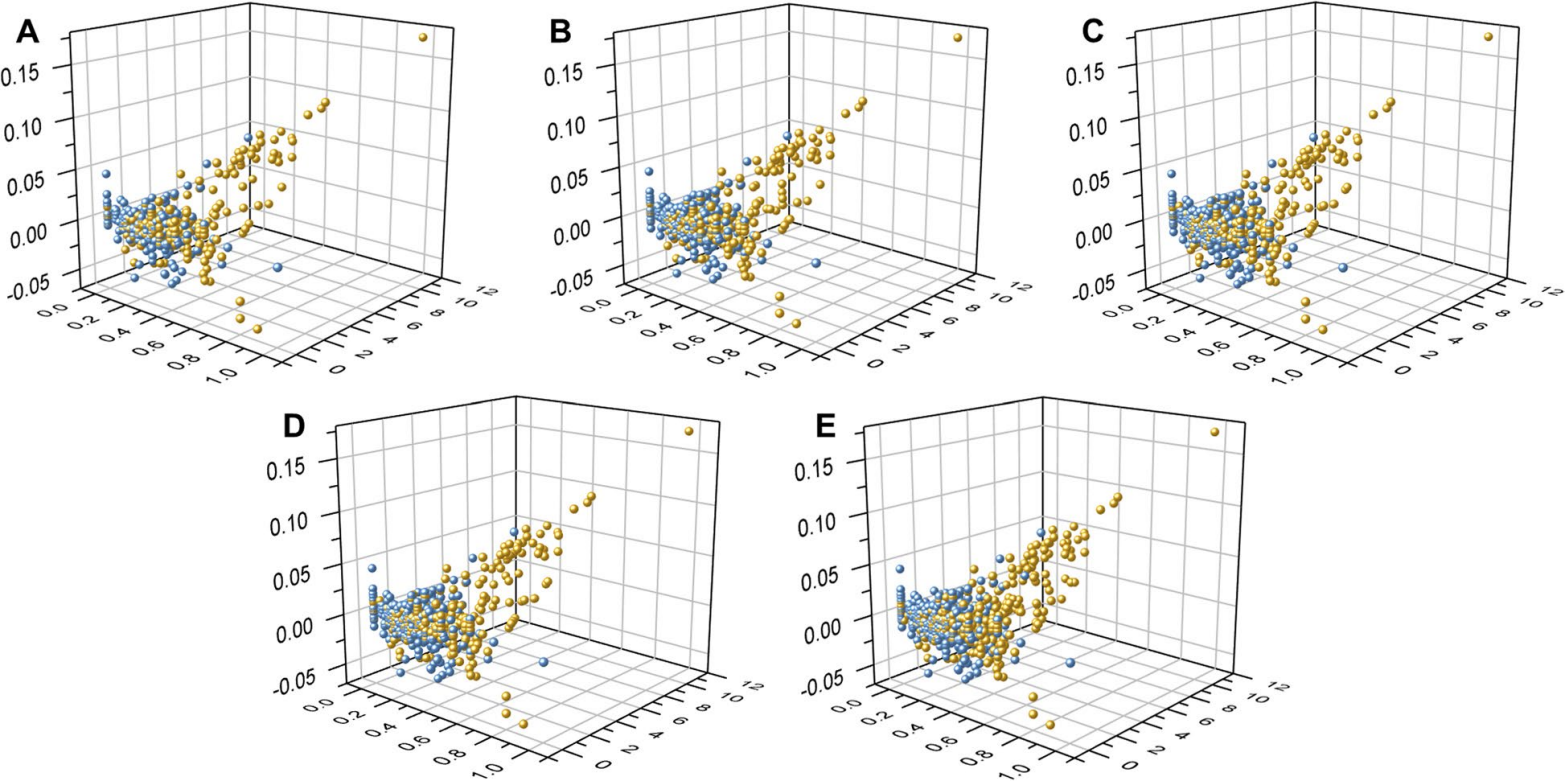
The data distribution with different augmentation ratios in feature space. **A** No data augmentation. **B** Proportion reaches 25%. **C** Proportion reaches 50%. **D** Proportion reaches 75%. **E** Proportion reaches 100%

**Fig. 8:**
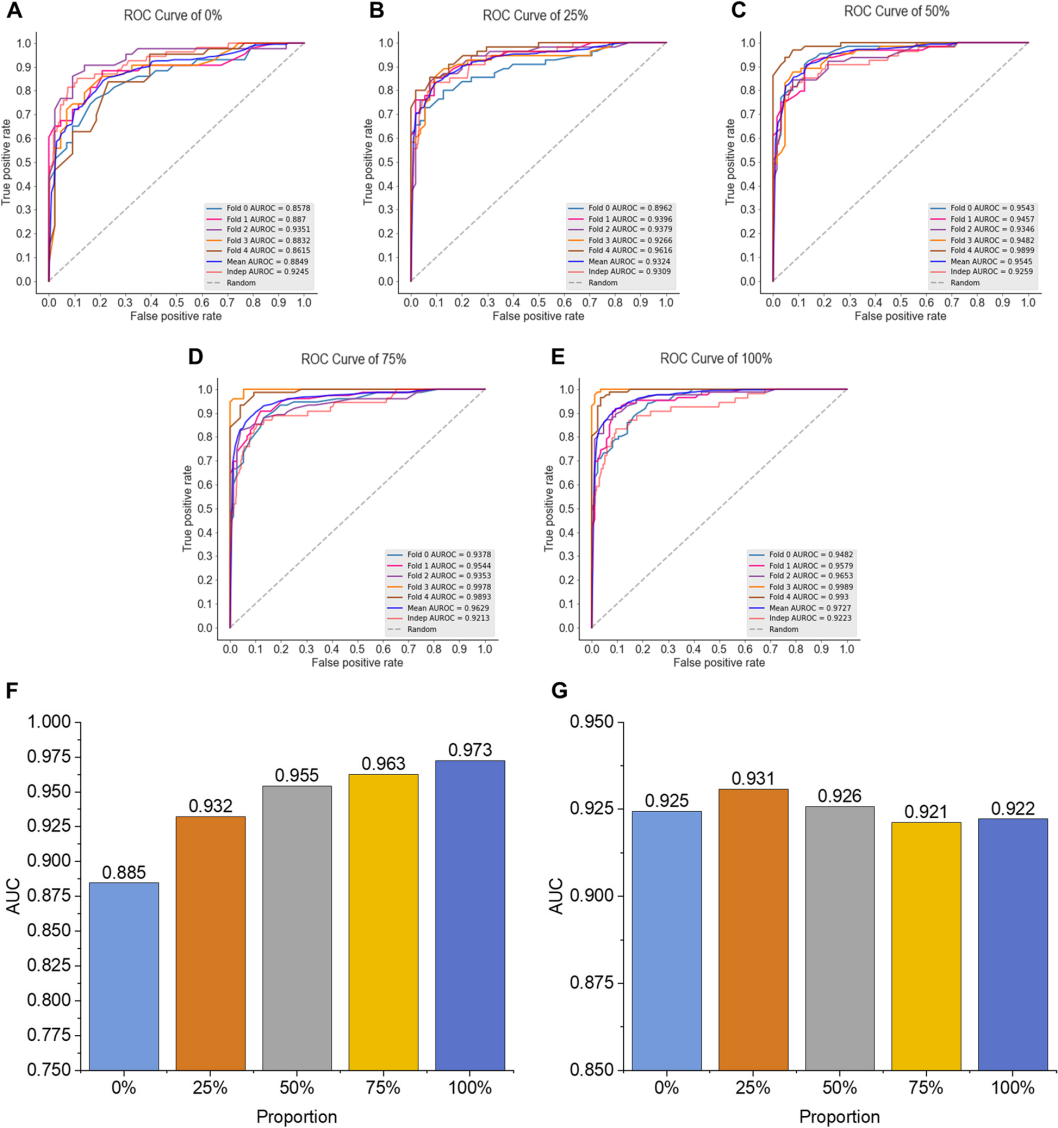
The prediction results on different proportions. **A** ROC curve of 0%. **B** ROC curve of 25%. **C** ROC curve of 50%. **D** ROC curve of 75%. **E** ROC curve of 100%. **F** Details of evaluation on training set. **G** Details of evaluation on independent test set

In this study, we employed a data augmentation method combining under-sampling and oversampling to process training set. We first qualitatively the distribution of three representative features (CTD_23, QSO_1, and APAAC_20) in three-dimensional space to observe the effect of data augmentation. As shown in Fig. 7, although there are obvious differences in feature distribution between positive and negative samples, the impact of changes in the data augmentation ratio on the feature space is not intuitive. Therefore, we further examined the impact of different augmentation proportions on model performance (Table 2). Details of evaluation for different algorithms are shown in Fig. 8.

The quantitative results are shown in Table 2 and Fig. 8. On the training set, the prediction performance of the model increases with the increase of the data augmentation ratio, indicating that larger data size brings more informative features, thereby producing better predictive ability. In addition, we found that when the data augmentation ratio is 25%, the model’s performance reaches a plateau on the independent set (AUROC = 0.931). As the data augmentation ratio further increases, the prediction ability fluctuates. These results indicated that the data augmentation strategy is effective under a wide range of thresholds and that choosing an appropriate threshold is helpful to improve the prediction performance of the model.

**Table 2:** Performance of data augmentation results with different proportions

Evaluation strategy	Proportion	*Sn*	*Sp*	*ACC*	*MCC*	AUROC
Training set validation	0%	0.800	0.819	0.809	0.620	**0.885**
	25%	0.853	0.839	0.846	0.695	**0.932**
	50%	0.863	0.876	0.870	0.741	**0.955**
	75%	0.904	0.904	0.904	0.810	**0.963**
	100%	0.912	0.909	0.910	0.823	**0.973**
Independent set validation	0%	0.852	0.822	0.824	0.454	**0.924**
	25%	0.833	0.861	0.858	0.497	**0.931**
	50%	0.852	0.884	0.882	0.549	**0.926**
	75%	0.833	0.899	0.893	0.566	**0.921**
	100%	0.815	0.907	0.899	0.569	**0.922**

The most important indicators are shown in bold

### Prediction performance comparison with existing models

**Fig. 9:**
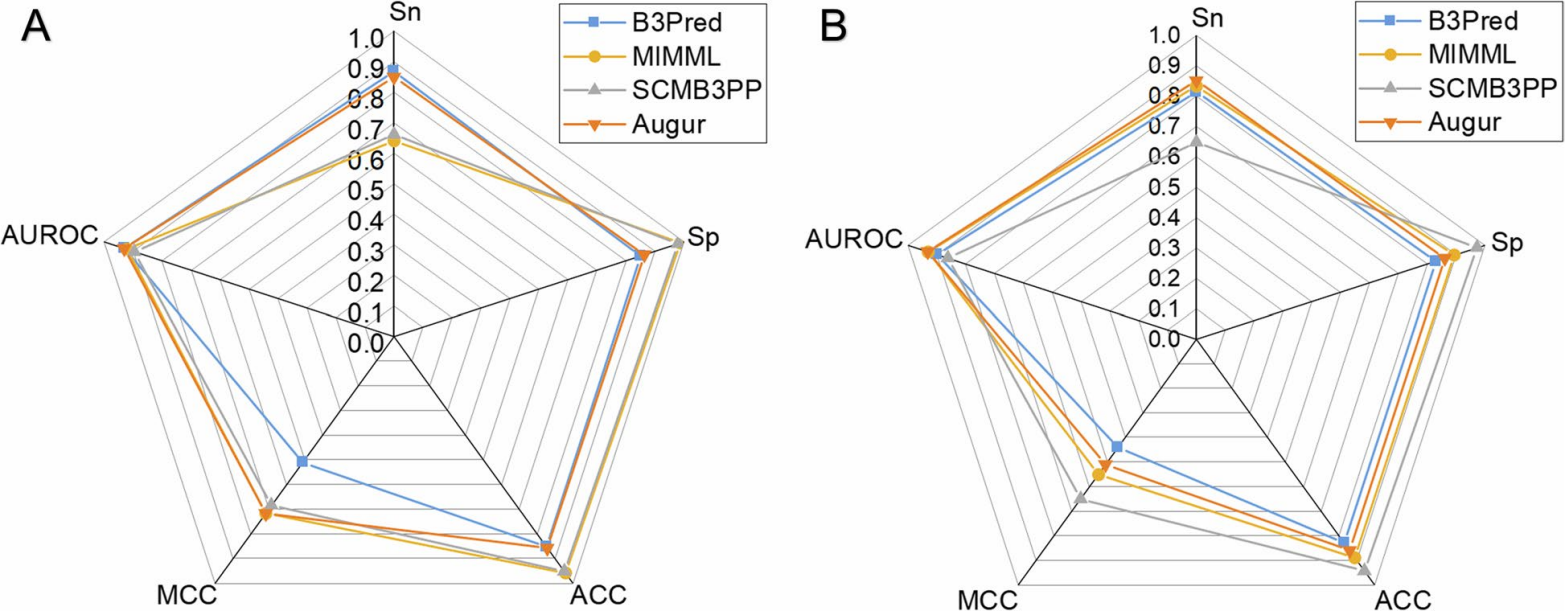
Radar plot for comparing Augur with other published models using (**A**) training set and (**B**) independent test set

To further examine the model’s predictive capability, we compared Augur with existing prediction tools using training set and independent data set. However, most of the models used different training data or did not provide standalone tools or web server, thereby making it difficult to provide a direct comparison. To solve it, we only chose three representative machine learning-based tools that are B3Pred, MIMML, and SCMB3PP [15, 16, 18]. For a fair and stringent comparison, we rebuilt the models of these three tools, and the corresponding performances were obtained. The predictive performances are shown in Table 3 and Fig. 9. We noticed that Augur is superior to other three predictors. Specifically, the AUROC of Augur is 0.932, which is 0.002, 0.010, and 0.036 higher than B3Pred, MIMML, and SCMB3PP on the training set, respectively. Furthermore, on the independent test set, the AUROC of Augur achieved an AUROC of 0.931, which is 0.031, 0.001, and 0.069 higher than B3Pred, MIMML, and SCMB3PP, respectively. These results indicated that Augur has superior predictive ability compared to existing tools.

**Table 3:** Performance comparison of Augur with the existing methods

Evaluation strategy	Classifier	*Sn*	*Sp*	*ACC*	*MCC*	AUROC
Training set validation	B3Pred	0.869	0.850	0.852	0.510	0.930
	MIMML	0.641	0.989	0.957	0.716	0.922
	SCMB3PP	0.661	0.980	0.951	0.684	0.896
	Augur	0.853	0.839	0.846	0.695	**0.932**
Independent set validation	B3Pred	0.814	0.830	0.829	0.440	0.900
	MIMML	0.833	0.894	0.889	0.550	0.930
	SCMB3PP	0.648	0.974	0.944	0.650	0.862
	Augur	0.833	0.861	0.858	0.497	**0.931**

Best performance metrics are shown in bold

## Discussion

The discovery of B3PPs provides an effective drug delivery solution for CNS treatment. B3PPs have the advantages of ordinary protein pharmaceutical preparations and small molecule pharmaceutical preparations. Moreover, B3PPs can directly combine with some bioactive proteins, offering convenience for related drug development and demonstrating substantial clinical treatment potential.

The existing B3PPs prediction models that are primarily based on imbalanced datasets can indeed lead to compromised generalizability. Such models may exhibit cases where *Sp* is much higher than *Sn*, as with MIMML and SCMB3PP. They may produce suboptimal results on datasets with inconsistent distributions. The disparity between *Sp* and *Sn* values suggests that these models, trained on datasets where negative samples significantly outnumber positive samples, struggle to accurately identify potential B3PPs, including misclassifying them as non-B3PPs.

Therefore, in this study, we investigated feature extraction methods, feature selection techniques, model construction methods, and data augmentation for the classification prediction problem of B3PPs. Firstly, we selected seven feature extraction methods and used the IG algorithm to select the key features. Then, we employed five machine learning algorithms and compared their performance, and the experimental results demonstrated that the RF algorithm was more suitable for constructing the B3PPs classification model. Next, we applied data augmentation techniques to process the B3PPs sequence data. Through comparative experiments, we demonstrated that this approach helps in building a highly accurate and generalizable prediction model. Finally, we proposed a new B3PPs prediction model named Augur. The comprehensive performance of this model was better than existing models, providing biologists with more directions for B3PPs research. The superior predictive performance of Augur can be attributed to two major factors: (i) properly processing the original training set addressing the issue of imbalanced positive and negative samples and (ii) choosing an appropriate data augmentation ratio helps the model achieve optimal predictive performance.

The prediction model for B3PPs holds significant implications for the design of peptide pharmaceuticals. It has the capability to forecast which peptides can penetrate the BBB, providing crucial guidance for the development of drugs targeting neurological disorders. By employing computational methods to screen potential peptide candidates, Augur can notably reduce the time and cost of experimental testing, thereby enhancing the efficiency of drug development. Furthermore, this model incorporates various sequence feature extraction methods that consider the physicochemical properties of peptides, aiding in the deeper understanding of structural and physicochemical characteristics that influence permeability through the BBB, which is a guide for the rational design of peptide drugs that are more effective in entering the brain. Continual development and refinement of these prediction models are a focal point in current research and have the potential to revolutionize the treatment strategies for various central nervous system diseases.

However, the lack of sufficient quality and robustness in data-sharing practices remains a key obstacle to the positive impact of machine learning models in the field of peptide and peptidomimetic drug discovery. Insufficient data quality may lead to poor generalization of models. Data harmonization, through techniques such as domain knowledge transfer, plays a crucial role in improving data quality and utilization for peptide identification. Among them, advanced algorithms such as interpretable generative models, few-shot generative models, and multimodal generative models will provide new solutions for peptide identification and drug discovery.

## Conclusions

The discovery of B3PPs marks a significant advancement in CNS drug delivery, combining the benefits of protein and small molecule therapeutics. However, existing B3PPs prediction models suffer from limited generalizability due to imbalanced datasets. This study addresses these challenges by exploring feature extraction, selection techniques, and data augmentation, with the RF algorithm emerging as the most suitable for B3PPs classification. The newly developed Augur model demonstrates superior performance in predicting B3PPs, offering valuable insights for drug development targeting neurological disorders. This breakthrough may enhance the efficiency of peptide-based drug discovery and pave the way for innovative treatment strategies for CNS diseases.

## Methods

### Benchmark dataset construction

In existing databases related to B3PPs, Brainpeps contains a collection of 259 different B3PPs sequences [4], while SATPdb includes 154 usable data [20]. Notably, B3Pdb boasts the largest and most comprehensive collection of B3PPs sequence data, with a total of 465 unique B3PPs sequences [1]. Therefore, we utilized the dataset provided by B3Pdb, comprising 269 unique B3PPs sequences and 2690 non-B3PPs sequences.

Since the limited number of positive samples and the imbalance between positive and negative samples are not conducive to building a robust prediction model, we therefore adopt a strategy that combines Random Under-Sampling (RUS) and Borderline SMOTE oversampling for data augmentation [21, 22]. Borderline SMOTE specifically targets minority class samples located at the borderline between majority and minority classes, rather than all minority class samples [22]. This approach minimizes the risk of introducing noise into the dataset and strengthens the classification boundary. In the classification problem of B3PPs, focusing on borderline samples enhances the model’s sensitive to these challenging boundary areas, thereby improving the accuracy of classifying minority class instances. Moreover, as Borderline SMOTE exclusively processes borderline samples, it also mitigates the risk of model overfitting. Specifically, we utilized the Borderline SMOTE algorithm at different thresholds (25%, 50%, 75%, and 100%) to oversample the positive samples. Meanwhile, we used RUS to reduce the negative samples, generating corresponding subsets of negative samples at each threshold to match the number of positive samples, as shown in Table 4.

**Table 4:** Dataset size with different augmentation thresholds

Augmentation threshold	Sample of positive data	Sample of negative data
0%	215	215
25%	273	273
50%	323	323
75%	376	376
100%	430	430

### Feature extraction methods

Adopting efficient feature extraction methods is a key step in building high-performance predictors [23–28]. Here, we chose seven feature extraction methods to formulate B3PPs sequences.

### Amino acids composition (AAC)

The AAC descriptor is utilized to measure the frequency of each natural amino acid in a peptide sequence, represented by {* A*, *C*, *D*, *E*, *F*, *G*, *H*, *I*, *K*, *L*, *M*, *N*, *P*, *Q*, *R*, *S*, *T*, *V*, *W*, *Y*} [29], which contains a total of 20 types. This feature is widely used in the construction of various peptide prediction models [30–32]. It is calculated as following:

1 $$\textit{f}\mathit{\left(\text{t}\right)}\textit{=}\frac{\textit{N}\mathit{\left(\text{t}\right)}}{\textit{L}}\mathit\,\textit{t}\mathit\in\mathit{\left\{{A,C,\dots\dots,Y}\right\}}$$

where ${\text{N}}\left({\text{t}}\right)$ is the number of amino acid type *t* and $L$ is the length of peptide. In this study, AAC generated a total of 20 features.

### Composition of k-spaced amino acid p×airs (CKSAAP):

The CKSAAP descriptor is used to calculate the frequency of amino acid pairs with *k* residue intervals [30, 33–35]. The base pairs include: {*AA, AC, …, YY*}. The feature is described as follows:

2 $$\begin{array}{cc}\textit{f}\mathit{\left(\text{pair}\right)}\textit{=}\frac{\textit{N}\mathit(\textit{pair}\mathit)}{{\textit{N}}_\textit{total}}&\mathit p\mathit a\mathit i\mathit r\mathit\in\mathit\{\mathit A\mathit A\mathit,\mathit A\mathit C\mathit,\mathit\dots\mathit\dots\mathit,\mathit Y\mathit Y\mathit\}\mathit,\end{array}$$

3 $${\textit{N}}_\textit{total}\mathit=L\mathit-k\mathit-\mathit1$$

where ${\text{pair}}$ represents the amino acid residue pair, ${\text{N}}({\text{pair}})$ is the corresponding occurrence count of the amino acid residue pair, ${\text{L}}$ represents the length of peptide chain, and ${\text{N}}_{\text{total}}$ is related to the parameter *k*. Given that the length of B3PPs peptide sequence is between 5 and 30 amino acids, the parameter *k* was set to 3. In this study, CKSAAP generated a total of 1600 features.

### Dipeptide deviation from expected mean (DDE)

The DDE descriptor mainly considers the occurrence frequency of a set of known dipeptides in a given polypeptide sequence. DDE is calculated based on three different parameters: dipeptide composition (${\text{D}}_{\text{c}}$), theoretical mean (${\text{T}}_{\text{m}}$), and theoretical variance (${\text{T}}_{\text{v}}$) [36, 37]. The calculation formula for ${\text{D}}_{\text{c}}$ is shown as following:

4 $$D_c\;\left(r,s\right)\;=\;\frac{N\left(r,s\right)}{L-1}\;r,s\;\in\;\left\{A,\;C\;\dots\dots,\;Y\right\}$$

where ${\text{L}}$ is the length of peptides and ${\text{r}}$ and $s$ represent two amino acids, respectively. The calculation formula for ${\text{T}}_{\text{m}}$ is given as:

5 $${T_m}\left({r,s}\right)=\frac{C_r}{C_N}\times\frac{C_s}{C_N} \quad\quad {r,s}\in\left\{A,\quad{C},......,{Y}\right\},$$

where ${\text{C}}_{\text{r}}$ is the number of codons that encoding amino acid type *r*, ${\text{C}}_{\text{s}}$ is the number of codons that encoding amino acid type *s*, and ${\text{C}}_{\text{N}}$ represents the total number of all the codons. The calculation formula for ${\text{T}}_{\text{v}}$ is given by:

6 $$T_v(r,s)=\frac{{T_m}\left({rs}\right)\times\left(\text{1} -T_m{(rs)}\right)}{{L}-{1}} \, \quad{r,s}\in\left\{{A},\quad{C},......,\quad{Y}\right\},$$

And finally, ${\text{DDE}}\left({\text{r}}\text{,} \, {\text{s}}\right)$ is calculated as:

7 $$DDE(r,s)=\frac{D_{c}(r,s)-T_{m}(r,s)}{\sqrt{T_{v}(r,s)}}\quad\quad{r,s}\in\left\{A,\quad{C},......,\quad{Y}\right\}$$

In this study, DDE generated a total of 400 features.

### Amphiphilic pseudo-amino acid composition (APAAC)

On the basis of the concept of pseudo-amino acid composition, APAAC adds physicochemical properties such as hydrophilicity and charge properties to encode sequence features [38]. Specifically, the hydrophobicity and hydrophilicity of the standardized polypeptide sequence are represented by ${\text{H}}_{1}\left({\text{i}}\right)$ and ${\text{H}}_{2}\left({\text{i}}\right)$, respectively:

8 $$\textit{H}_\textit{i,j}^{\mathit1}\mathit{\left(\text{i}\right)}{\textit{=H}}_{\mathit1}\mathit{\left(\text{i}\right)}{\textit{H}}_{\mathit1}\mathit{\left(\text{j}\right)}\mathit,$$

9 $$\textit{H}_\textit{i,j}^{\mathit2}\mathit{\left(\text{i}\right)}{\textit{=H}}_{\mathit2}\mathit{\left(\text{i}\right)}{\textit{H}}_{\mathit2}\mathit{\left(\text{j}\right)}\mathit,$$

Accordingly, sequence order can be given as:

10 $$\left\{\begin{array}{l}{\tau}_{{2}{\text{k-1}}}\text{=}\frac{1}{\text{N-k}}\sum\limits _{\text{i=1}}^{\text{N} - \lambda}{\text{H}}_{\text{i,i+k}}^{1}\\ {\tau}_{\text{2k}}\text{=}\frac{1}{\text{N-k}}\sum\limits_{\text{i=1}}^{\text{N} - \lambda}{\text{H}}_{\text{i,i+k}}^{2}\end{array}\right.{1}\preceq{\text{k}}\preceq\lambda\text{,}$$

Finally, APAAC can be defined as:

11 $${\textit{P}}_\textit{u}\textit{=}\mathit{\left\{\begin{array}{l}\frac{{\text{f}}_\text{c}}{\sum_\text{i=1}^{20}{\text{f}}_\text{i}+\text{w}\sum_\text{j=1}^{2\lambda}\tau_\text{j}}\,1\leq\text{c}\leq20\\\frac{\text{w}\tau_\text{c}}{\sum_\text{i=1}^{20}{\text{f}}_\text{i}+\text{w}\sum_\text{j=1}^{2\lambda}\tau_\text{j}}\,20\text{+}1\leq\text{c}\leq20\text{+}2\lambda\end{array}\right.}$$

where ${\text{w}}$ is the weighting factor and it was set to 0.5 in this study and APAAC generated a total of 24 features.

### Adaptive skip dipeptide composition (ASDC)

The ASDC descriptor captures spacing-specific dipeptide component information by splitting the protein sequence into consecutive dipeptides and calculating the frequency of each specific jumping dipeptide in the protein sequence [39]. It can be defined as follows:

12 $$\text{ASDC} = \left(f_{v_1},f_{v_2},...f_{v_{400}}\right),$$

13 $${\textit{f}}_{{\textit{v}}_\textit{i}}\textit{=}\frac{\mathit\sum_\textit{g=1}^{\textit{L}\mathit-\mathit1}\textit{O}_\textit{i}^\textit{g}}{\mathit\sum_\textit{i=1}^{\mathit{400}}\mathit\sum_\textit{g=1}^{\textit{L}\mathit-\mathit1}\textit{O}_\textit{i}^\textit{g}}$$

where ${\text{g}}$ represents ${\text{g}}$-gap, which is the gap between residues. ${\text{f}}_{{\text{v}}_{\text{i}}}$ is the frequency of the type ${\text{i}}$ ${\text{g}}$-gap dipeptide, while ${\text{O}}_{\text{i}}^{\text{g}}$ represents its count of occurrence. In this study, ASDC generated a total of 400 features.

### Composition/transition/distribution (CTD)

The CTD descriptor was originally proposed by Dubchak et al., which considers unique structural and physicochemical properties in peptide sequences [40]. As ML techniques require fixed property vectors as input for classification, amino acids should be replaced by numeric symbols. These symbols were divided into three categories, including polar, neutral, and hydrophobic. Details about the division of the amino acids are provided in Table 5. Composition (C) is the percent for each encoded class in the sequence, which is defined as:

**Table 5:** Amino acid attributes and division of the amino acids to groups^24^

Property	Group 1	Group 2	Group 3
Charge	Neutral	Negatively charged	Positively charged
	A, C, F, G, H, I, L, M, N, P, Q, S, T, V, W, Y	D, E	K, R
Hydrophobicity	Hydrophobicity	Neutral	Polar
	C, F, I, L, M, V, W	A, G, H, P, S, T, Y	D, E, K, N, Q, R
Normalized van der Waals volume	0–2.78	2.95–4.0	4.03–8.08
	A, C, D, G, P, S, T	E, I, L, N, Q, V	F, H, K, M, R, W, Y
Polarity	4.9–6.2	8.0–9.2	10.4–13.0
	C, F, I, L, M, V, W, Y	A, G, P, S, T	D, E, H, K, N, Q, R
Polarizability	0–0.108	0.128–0.186	0.219–0.409
	A, D, G, S, T	C, E, I, L, N, P, Q, V	F, H, K, M, R, W, Y
Secondary structure	Coil	Helix	Strand
	D, G, N, P, S	A, E, H, K, L, M, Q, R	C, F, I, T, V, W, Y
Solvent accessibility	Buried	Intermediate	Exposed
	A, C, F, G, I, L, V, W	H, M, P, S, T, Y	D, E, K, N, R, Q

14 $$Composition= \frac{{N}_{s}}{L} \,s=1, 2, 3,$$

where ${\text{N}}_{\text{s}}$ is the number of *s* in the encoded sequence and ${\text{L}}$ is the total length of the encoded sequence. Transition (T) represents the percent frequency of one amino acid following by another in the encoded sequence, which is defined as:

15 $${\text{Transition}}= \text{ } \frac{{\text{N}}_{\textrm{st}}\text{+}{\textrm{N}}_{\text{ts}}}{{\textrm{L-1}}} \, {\textrm{st}} \, \text{=}\textrm{ (12),(13),(23),}$$

where ${\text{N}}_{\text{st}}$ and ${\text{N}}_{\text{ts}}$ are the number of dipeptides encoded as “st” and “ts,” respectively. Distribution (*D*) describes the distribution of each property in the sequence. There are five distribution descriptors for each property, including the position percent in the sequence for the first, 25%, 50%, 75%, and 100% residues, respectively. For each group, it is defined as:

16 $${D}_{x}=\frac{{P}_{i}}{L} (i=1, 25, 50, 75, 100;x=1, 2, 3, 4, 5),$$

where *P*_1_, *P*_25_, *P*_50_, *P*_75_, and *P*_100_ are the position of the first, 25%, 50%, 75%, and 100% residues occurrence, respectively. In this study, CTD generated a total of 273 features.

### Quasi-sequence-order (QSO)

QSO consists of two parts: the Grantham distance matrix and the Schneider–Wrede matrix. The Grantham distance matrix measures the biochemical property differences between different amino acids. The Schneider–Wrede matrix is used to calculate the physicochemical properties of peptide chains, such as charge distribution [41]. The QSO can be defined as follows:

17 $${\text{X}}_{\text{r}}\text{=}\left\{\begin{array}{l}\frac{{\text{f}}_{\text{r}}}{\sum_{\text{j=1}}^{20}{{\text{f}}}_{\text{j}}+ \text{w} \sum_{\text{q=1}}^{\varphi}\tau_{\text{q}}} \, {1}\leq{\text{r}}\leq{20}\\ \frac{{\text{w}}\tau_{\text{d-20}}}{\sum_{\text{j=1}}^{20}{{\text{f}}}_{\text{j}}+ \text{w} \sum_{\text{q=1}}^{\varphi}\tau_{\text{q}}} \, {20}\text{+}{1}\leq{\text{r}}\leq{20}\text{+}{\varphi}\end{array}\right.$$

where ${\text{f}}_{\text{r}}$ is the normalized frequency of the ${\text{r}}$ type amino acid, while ${\text{w}}$ is the weight factor that influences the sequence order effect (in this study, ${\text{w}}$ is set to 0.05), and $\varphi$=max{*L*-1}. Finally, QSO generated a total of 44 features.

### Feature selection

In order to eliminate noise and enhance computational efficiency, we adopted the information gain (IG) feature selection method to obtain optimal feature subset. IG measures the contribution of a feature to the classification task [42, 43]. A higher IG value indicates a larger amount of information and greater importance, which is beneficial for accurately performing classification tasks. Here, the information entropy can be defined as:

18 $${\text{H}}\left(\text{B|A}\right)\text{=-}\sum_{{\text{a}}\in{\text{A}},{\text{b}}\in{\text{B}}}{\text{p}}\left({\text{a}}\text{,} \, {\text{b}}\right){\text{log}}_{2}\frac{{\text{p}}\left({\text{a}}\text{,}\text{ b}\right)}{{\text{p}}\left({\text{a}}\right)},$$

where ${\text{H}}\left(\text{B|A}\right)$ is the information entropy of *B* when *A* holds, while *a* and *b* are the values of *A* and *B*, respectively. And *p* (*a*, *b*) is the probability of both *a* and *b* holding. Accordingly, the IG can be represented as the difference between the entropy of system ${\text{C}}$ and the information entropy of feature ${\text{X}}$:${\text{IG}}\left({\text{X}}\right)= \text{ H} \left({\text{C}}\right)\text{-H}\left(\text{C|X}\right)$

19 $$\text{=-}\sum_{\text{i=1}}^{\text{n}}{\text{P}}\left({\text{C}}_{\text{i}}\right)\times{{\text{l}}{\text{og}}}_{2}{\text{P}}\left({\text{C}}_{\text{i}}\right) + \text{P} \left({\text{x}}\right)\sum_{\text{i=1}}^{\text{n}}{\text{P}}\left({\text{C}}_{\text{i}}\text{|x}\right)\times{\text{log}}_{2}{\text{P}}\left({\text{C}}_{\text{i}}\text{|x}\right) + \text{P} \left(\stackrel{\mathrm{-}}{\text{x}}\right)\sum_{\text{i=1}}^{\text{n}}{\text{P}}\left({\text{C}}_{\text{i}}\text{|}\stackrel{\mathrm{-}}{\text{x}}\right)\times{\text{log}}_{2}{\text{P}}\left({\text{C}}_{\text{i}}\text{|}\stackrel{\mathrm{-}}{\text{x}}\right)$$

where ${\text{x}}$ is the feature ${\text{X}}$ appearing in the system, while $\stackrel{\mathrm{-}}{\text{x}}$ is the opposite.

### ML algorithms

#### Random forest

Random forest (RF) is an ensemble of multiple decision trees, each trained on randomly selected features and data subsets. To some extent, it can avoid overfitting and improve the accuracy and generalization ability of the model. The decision trees are trained independently and they are combined as the final result through voting or averaging. In this study, the implementation of the RF was conducted by the scikit-learn library [44], which can be installed by using instructions. We used five-fold cross-validation to assess the performance of models with different numbers of trees and used the grid search strategy to optimize the number of decision trees. In detail, the number of trees was set from 5 to 300. The criterion was set to “Gini,” indicating that Gini impurity is used as the quality measure for splits. The maximum number of features considered for splitting was set to the square root of the total number of features. After conducting the optimization, it has been observed that the model performs optimally when the number of trees is at 160.

### LightGBM

LightGBM is a ML algorithm based on Gradient Boosting Decision Trees (GBDT) [45]. Different from traditional GBDT, LightGBM improves training speed and accuracy by performing multiple sampling of data when training. It divides features into different subsets, where features within the same subset are usually mutually exclusive, to solve the problem of sparse high-dimensional data. Additionally, LightGBM uses the Leaf-Wise algorithm with depth constraints to construct decision trees, ensuring that trees will split at the node that maximally reduces the error. In this study, we used the grid search strategy to optimize the number of leaves and learning rate based on five-fold cross-validation test. The search range for the number of leaves was set from 20 to 100, and the depth range was set from 10 to 60. Meanwhile, the learning rate was set to be searched within the range of 0.01 to 0.15. After conducting the optimization, it was discovered that the model achieved the best performance when the number of leaves was set to 31 and the learning rate was set to 0.1.

### Logistic regression

Logistic regression (LR) algorithm models the relationship between input and output variables to predict the value of output variable. LR uses the sigmoid function to map the input variables to a probability value between 0 and 1. It transforms linear regression into logistic regression and utilizes maximum likelihood estimation to define the cost function for training. LR is commonly used as a base classifier in ensemble learning, where multiple classifiers are combined to form a powerful one. It can be achieved through voting or weighted averaging to achieve higher accuracy. In this study, the implementation of the LR was conducted by the scikit-learn library [44], which can be installed by using instructions. We constructed the model using the L2 regularization algorithm and chose the L-BFGS algorithm for optimizing the model’s parameters. Furthermore, we also implemented five-fold cross-validation to assess the performance of the model.

### K-nearest neighbor

The K-nearest neighbor (KNN) algorithm is a classic classification algorithm based on distance measurement. KNN determines the *K*-nearest neighbors by computing the distances between known data points and the new data point and then predicts the classification of the new data point. In this study, the implementation of the KNN was conducted by the scikit-learn library [44], and the grid search strategy was employed to optimize the *K*-value. Ultimately, it was found that the model achieved better results when the *K*-value was set to 3.

### Support vector machine

The support vector machine (SVM) algorithm is a linear classification method based on maximum margin classification [46]. SVM is capable of mapping input data into a high-dimensional space and identifying a decision boundary. The data points closest to this boundary are referred to as support vectors, while the distance from the data points to the boundary is referred to as the margin. In this study, the implementation of the SVM was conducted by the open source software library LIBSVM developed by Chang and Lin, which can be downloaded from the website (https://www.csie.ntu.edu.tw/~cjlin/libsvm/) [47]. We chose the poly kernel function to obtain the classification hyperplane. We conducted the grid search strategy to optimize the regularization parameter *C* within the range of 0.1 to 15 and the kernel parameter gamma within the range of 0.001 to 10 based on five-fold cross-validation.

### Model evaluation metrics

Cross-validation is a statistical analysis method for evaluating model performance [48, 49]. In order to save computational time, the five-fold cross-validation was used to estimate the performance of the proposed method. We used Sensitivity (*Sn*), Specificity (*Sp*), Matthews correlation coefficient (*MCC*), F1 score (F1), and Accuracy (*ACC*) to assess the predictive capability of the model [50–52].

20 $${\text{Sensitivity =}}\frac{\text{TP}}{\text{TP+FN}}$$

21 $${\text{Specificity = }}\frac{\textrm{TN}}{\text{TN+FN}}$$

22 $${\text{MCC = }}\frac{\text{TP}\times\text{TN}-\textrm{FP}\times\text{FN}}{\sqrt{\left(\text{TP+FP}\right)\left(\text{TP+FN}\right)\left(\text{TN+FP}\right)\left(\text{TN+FN}\right)}}$$

23 $$\text{F1} = \frac{2\times{\text{Sensitivity}}\times{\text{Precision}}}{{\text{Sensitivity}}+{\text{Precision}}}$$

24 $$\text{ACC} = \frac{\text{TP+TN}}{\text{TP+TN+FP+FN}}$$

where TP is true positive and FN is false negative, they represent the number that the B3PPs are predicted as B3PPs and non-B3PPs, respectively. On the contrary, TN is true negative and FP is false positive; they represent the number that the non-B3PPs are predicted as B3PPs and non-B3PPs, respectively.

In addition, we also calculated the area under the receiver operating characteristic curve (AUROC) to objectively evaluate the proposed model. The AUROC ranges from 0 to 1 and the higher the AUROC the better the prediction performance is [53–55].

## Data Availability

All code and data generated or analyzed during this study are included in this published article, its supplementary information files, and publicly available repositories: figshare (https://doi.org/10.6084/m9.figshare.25466461.v4 [56].

## Abbreviations

CNS: Central nervous system
BBB: Blood–brain barrier
B3PPs: Blood–brain barrier penetrating peptides
ML: Machine learning
RUS: Random Under-Sampling
AAC: Amino acids composition
CKSAAP: Composition of k-spaced amino acid pairs
DDE: Dipeptide deviation from expected mean
APAAC: Amphiphilic pseudo-amino acid composition
ASDC: Adaptive skip dipeptide composition
CTD: Composition/transition/distribution
QSO: Quasi-sequence order
IG: Information gain
RF: Random forest
GBDT: Gradient boosting decision trees
LR: Logistic regression
KNN: K-nearest neighbor
SVM: Support vector machine
MCC: Matthews correlation coefficient
AUROC: Area under the receiver operating characteristic curve
TSL: Two Sample Logo
